# Supplementary material for: LEOPARD: missing view completion for multi-timepoint omics data via representation disentanglement and temporal knowledge transfer
Source: Nat Commun. 2025 Apr 6;16:3278. doi: 10.1038/s41467-025-58314-3 (PMC11972361; doi:10.1038/s41467-025-58314-3)
Supplement: Supplementary file 1 — Supplementary Information [file 41467_2025_58314_MOESM1_ESM.pdf]

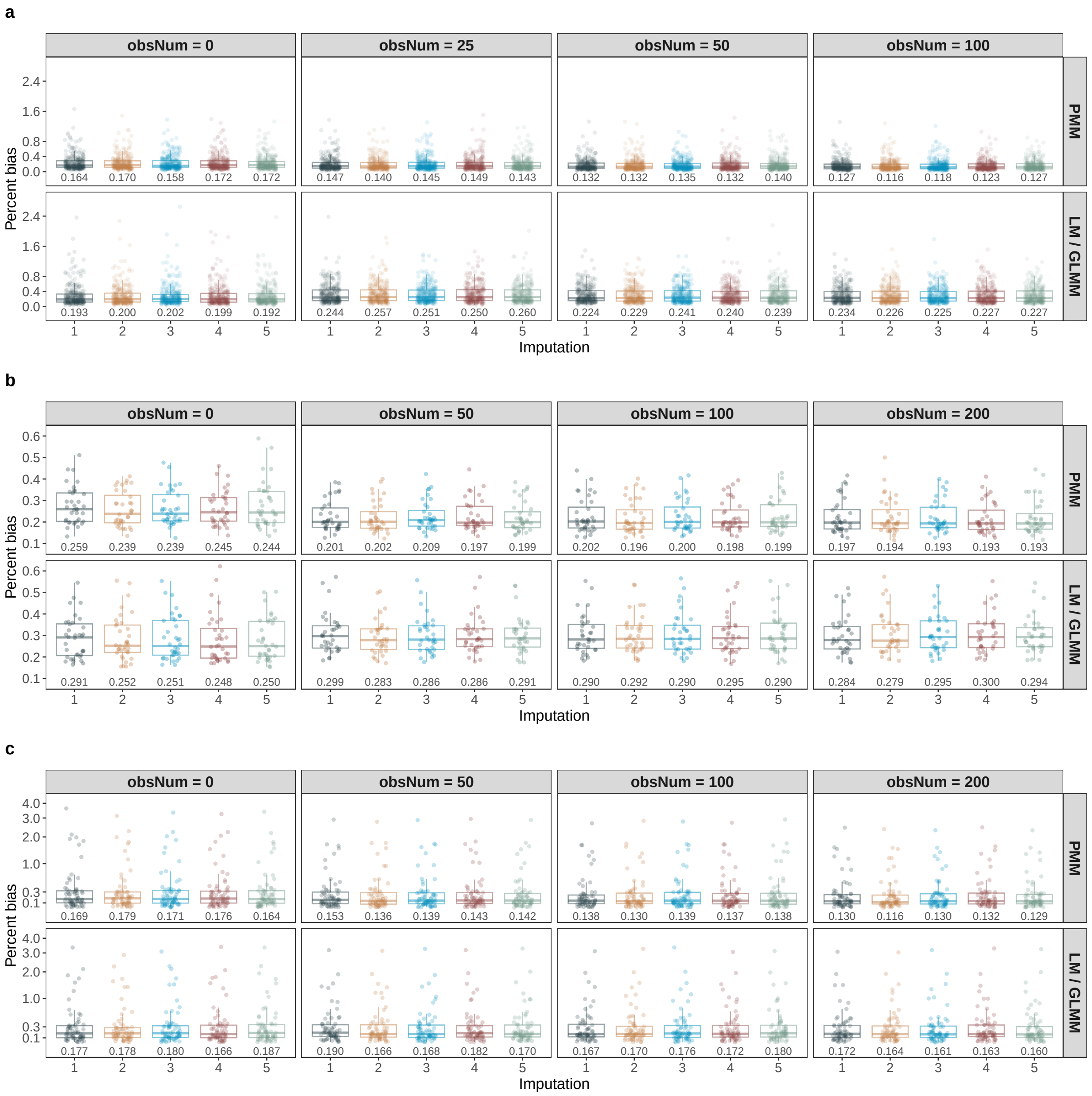

Supplementary Figure 1 Percent bias of each individual imputation of the multiple imputation methods PMM, LM, and GLMM calculated on $\mathcal{D}^{\mathrm{test}}_{v=\mathrm{v2},t=\mathrm{t2}}$ of the MGH COVID proteomics dataset (**a**), KORA metabolomics dataset (**b**), and KORA multi-omics dataset (**c**), with varying obsNum. **a-c**, imputation is performed five times ($m = 5$) for each method. The x-axis indicates each individual imputation. Each dot represents a percent bias value for a variable. The value below each box represents the median. Please note that LM is used for imputation instead of GLMM when obsNum $= 0$. Source data are provided as a Source Data file.

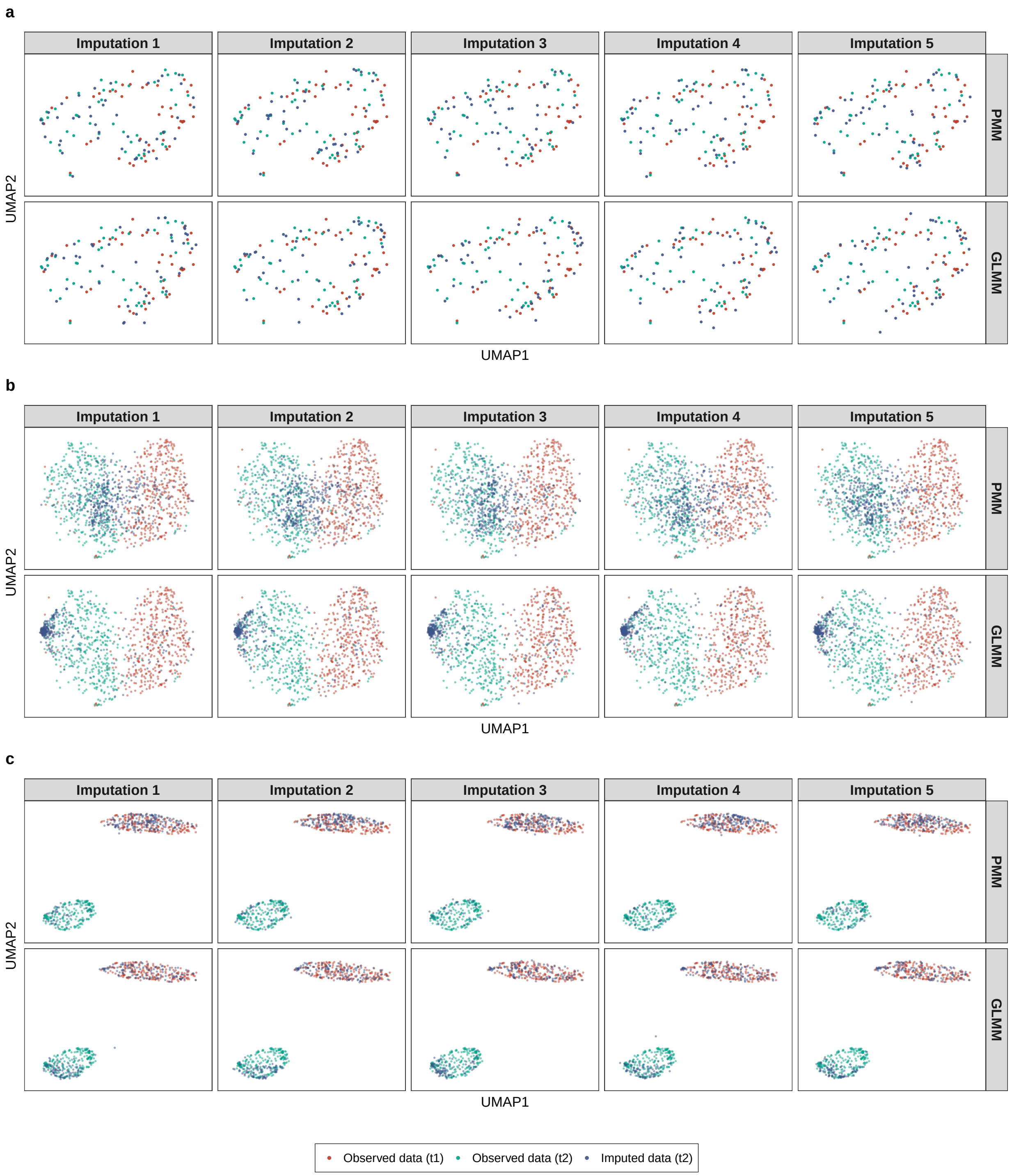

Supplementary Figure 2 UMAP representations of each individual imputation (Imputation 1 to 5) of methods PMM and GLMM and corresponding observed data of three benchmark datasets. **a-c**, UMAP models are initially fitted with the training data from the MGH COVID proteomics dataset (**a**, t1: D0, t2: D3), KORA metabolomics dataset (**b**, t1: F4, t2: FF4), and KORA multi-omics dataset (**c**, t1: S4, t2: F4). Subsequently, the trained models are applied to the corresponding observed data (represented by red and green dots for t1 and t2) and each individual imputation of PMM and GLMM (represented by blue dots) under the setting of obsNum = 100 for the MGH COVID dataset and obsNum = 200 for the two KORA-derived datasets. Source data are provided as a Source Data file.

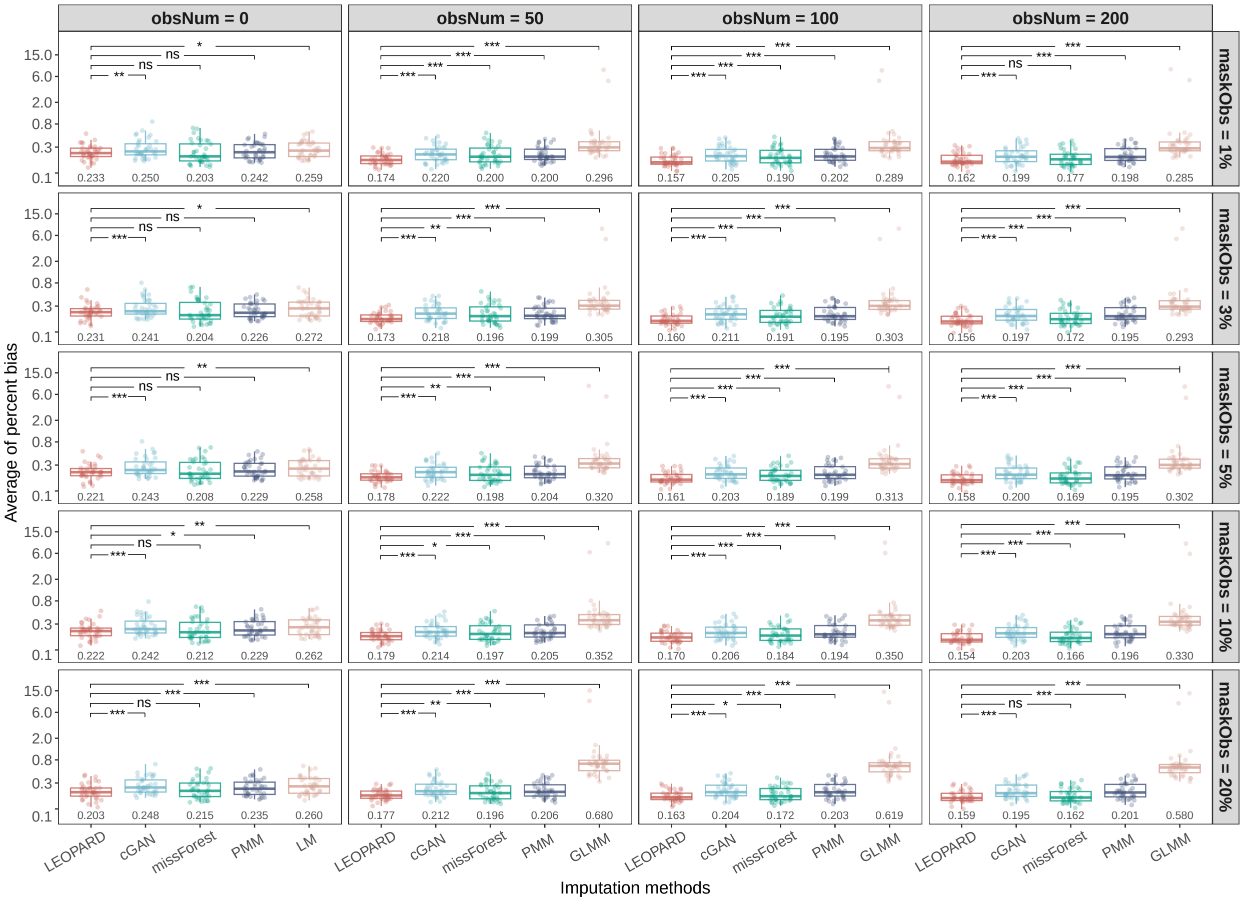

Supplementary Figure 3 The average PB are computed for $\mathcal{D}^{\text{test}}_{v=v2,t=t2}$ of the KORA metabolomics dataset across 10 repeated completions, under each maskObs. In each repetition, the data points are masked randomly. Each dot represents a PB value for a variable. The value below each box represents the median. Please note that LM is used instead of GLMM when obsNum = 0. Source Data are provided as a Source Data file.

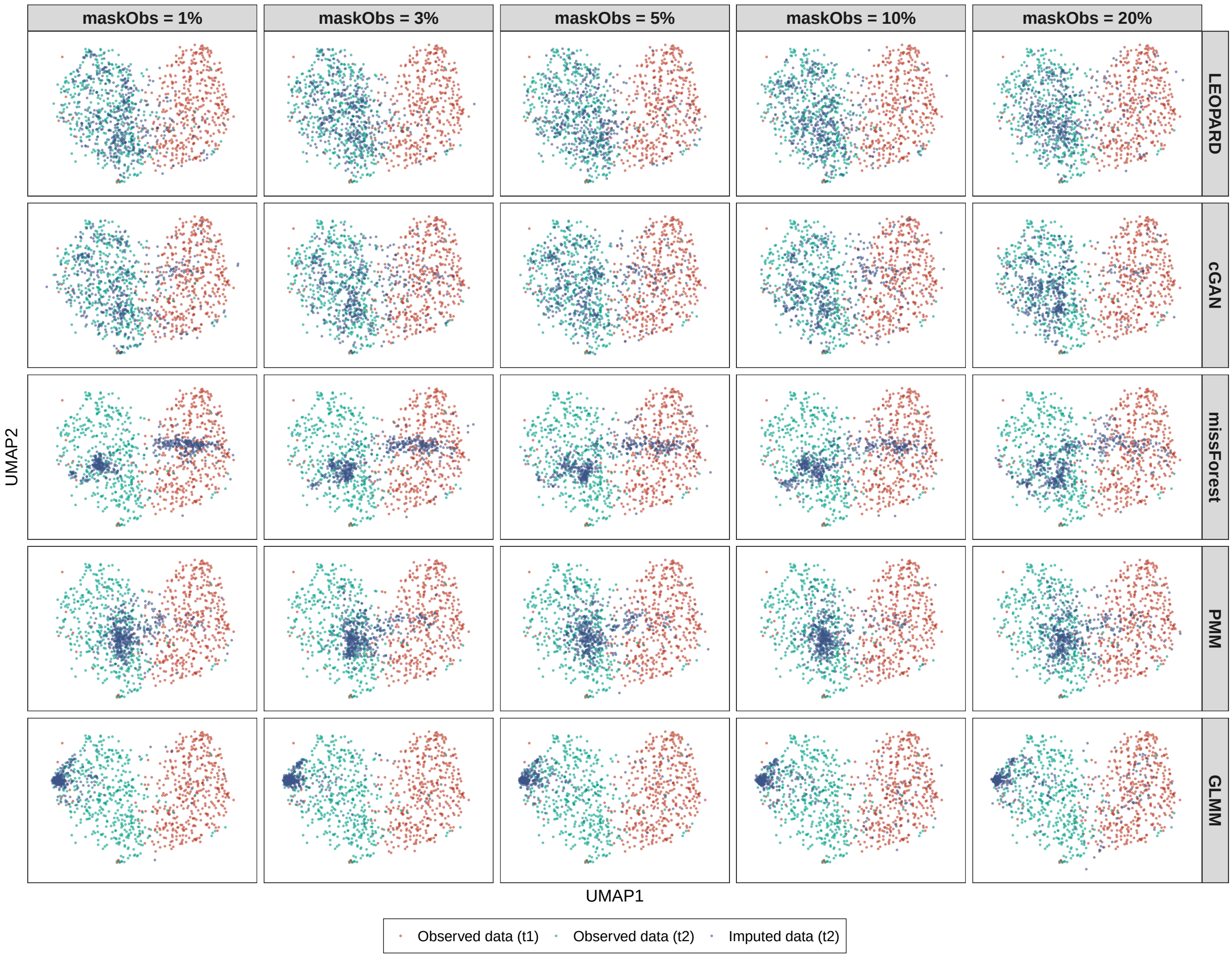

Supplementary Figure 4 UMAP models are initially fitted with the training data from the KORA multi-omics dataset (t1: S4, t2: F4). Subsequently, the trained models are applied to the corresponding observed data (represented by red and green dots for t1 and t2) and the data imputed by different methods (represented by blue dots) under obsNum = 200 and varying maskObs. For each maskObs, only the repetition that exhibits the lowest median of PB is visualized. The distributions of red and green dots illustrate the variation across the two timepoints, while the similarity between the distributions of blue and green dots indicates the quality of the imputed data. A high degree of similarity suggests a strong resemblance between the imputed and observed data. Source data are provided as a Source Data file.

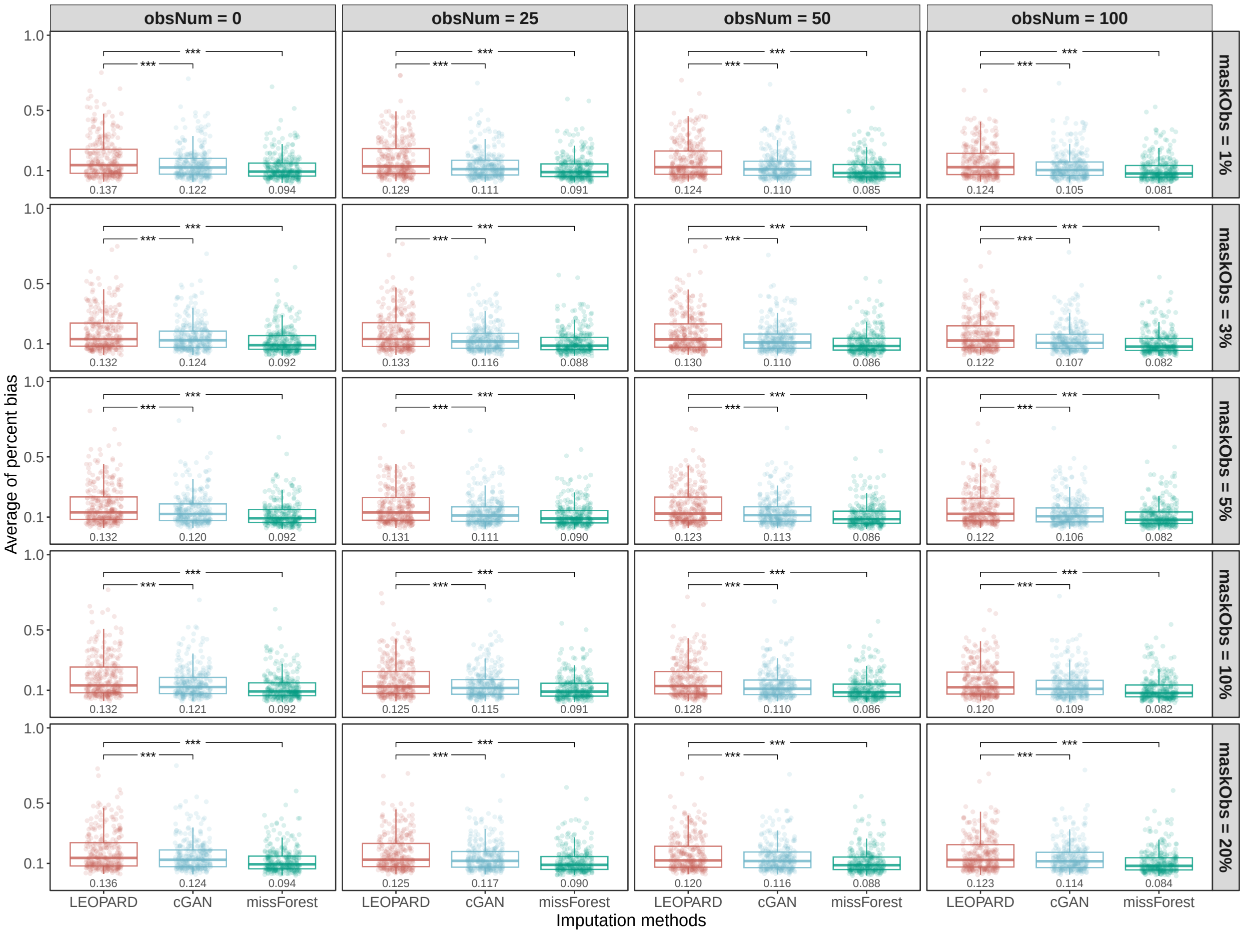

Supplementary Figure 5 The average percent bias are computed for $\mathcal{D}^{\mathrm{test}}_{v=\mathrm{v2},t=\mathrm{t2}}$ across 10 repeated completions, under each maskObs. In each repetition, the data points are masked randomly. Due to the exceptionally high computational demands of PMM and GLMM, these two methods are not evaluated on this dataset. Each dot represents a percent bias value for a variable. The value below each box represents the median. Source data are provided as a Source Data file.

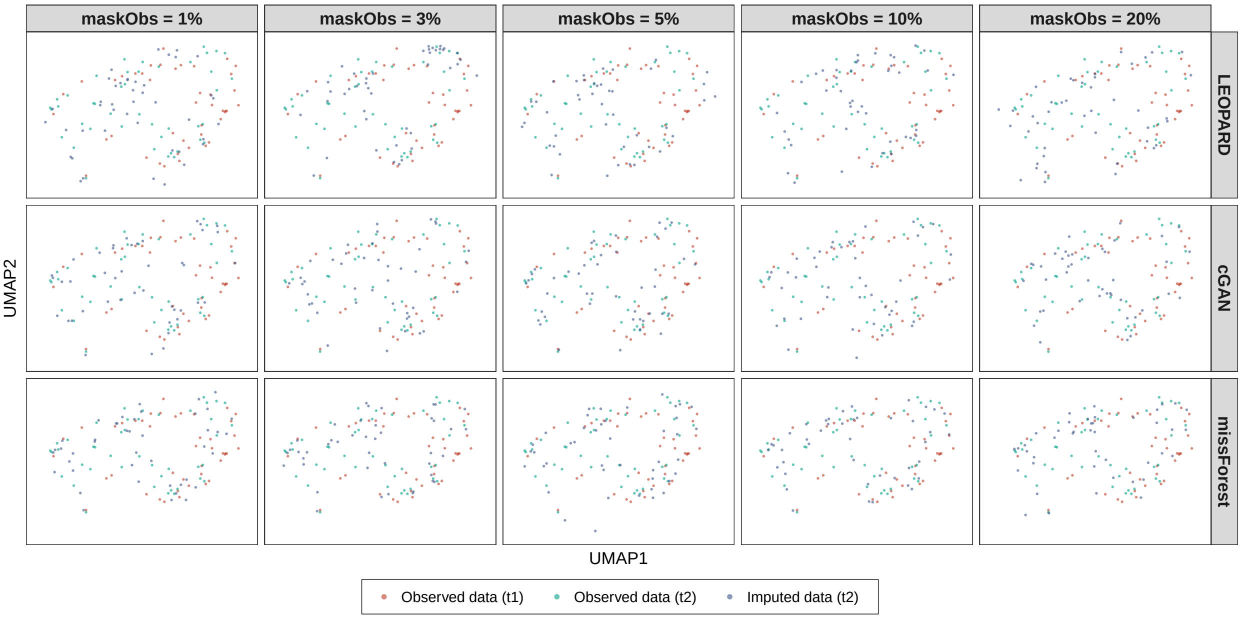

Supplementary Figure 6 UMAP models are initially fitted with the training data (t1: D0, t2: D3). Subsequently, the trained models are applied to the corresponding observed data (represented by red and green dots for t1 and t2) and the data imputed by different methods (represented by blue dots) under $obsNum = 100$ and varying maskObs. For each maskObs, only the repetition that exhibits the lowest median of percent bias is visualized. Source data are provided as a Source Data file.

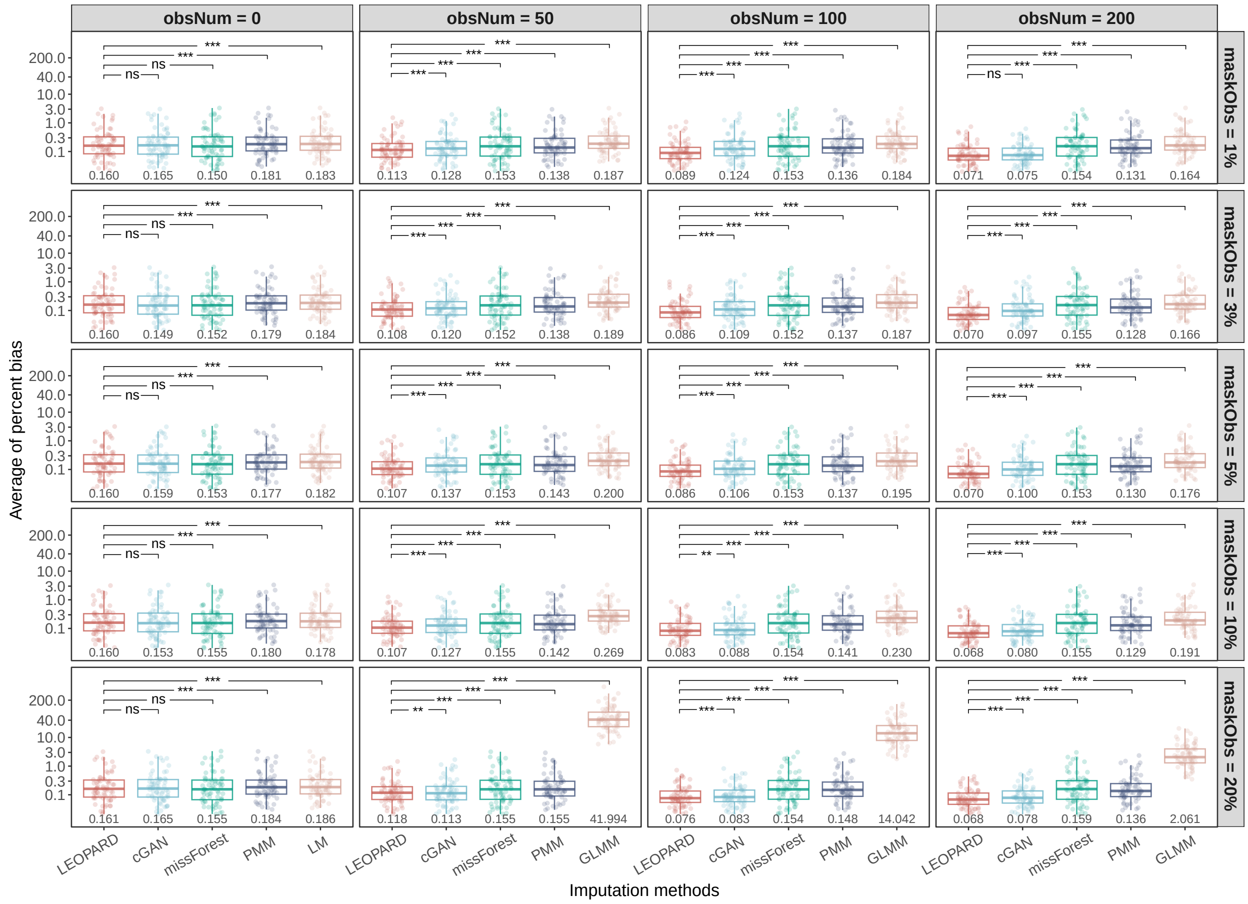

Supplementary Figure 7 The average percent bias are computed for $\mathcal{D}^{\text{test}}_{v=v2,t=t2}$ across 10 repeated completions, under each maskObs. In each repetition, the data points are masked randomly. Each dot represents a percent bias value for a variable. The value below each box represents the median. Please note that LM is used instead of GLMM when obsNum = 0. Source data are provided as a Source Data file.

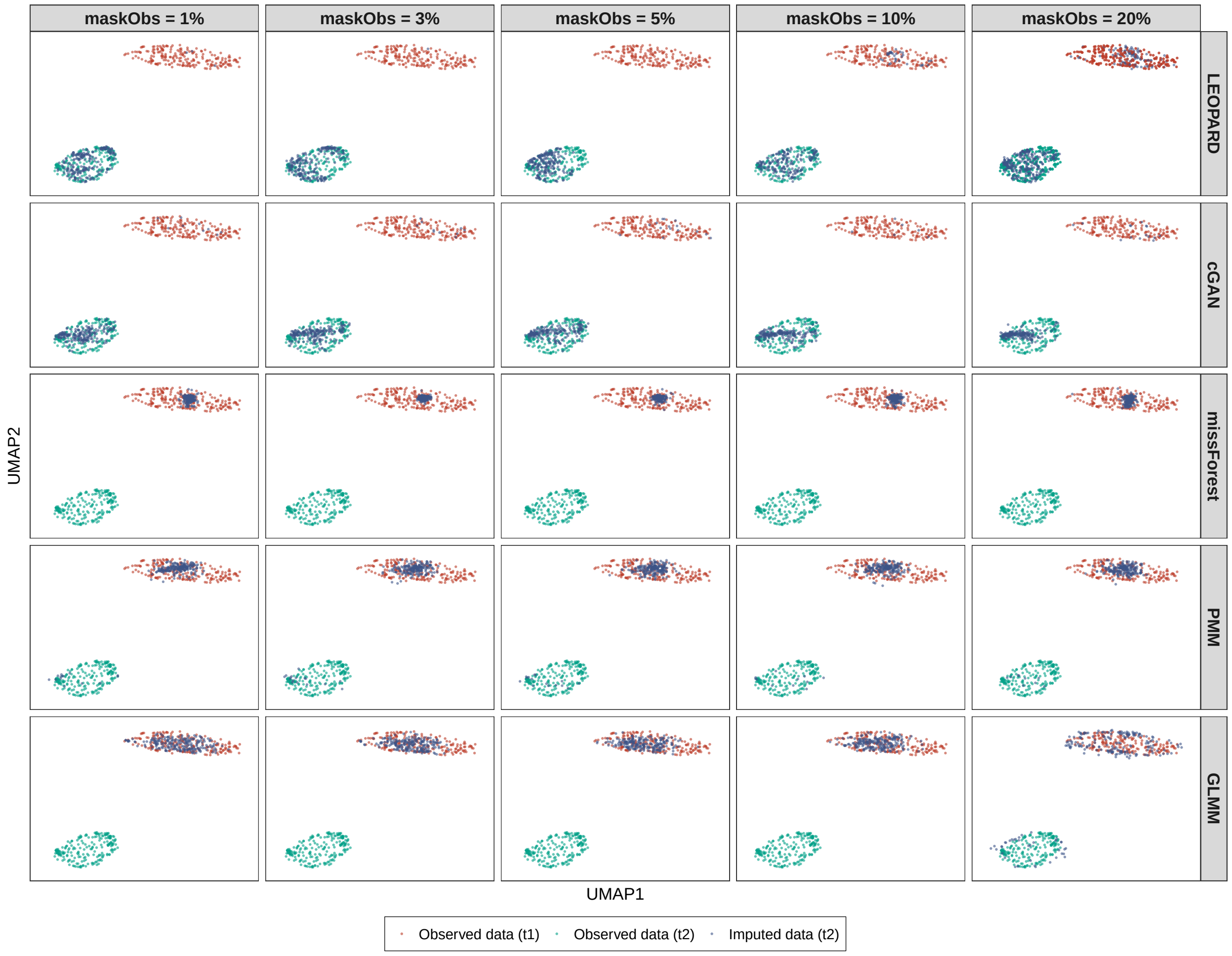

Supplementary Figure 8 UMAP models are initially fitted with the training data (t1: S4, t2: F4). Subsequently, the trained models are applied to the corresponding observed data (represented by red and green dots for t1 and t2) and the data imputed by different methods (represented by blue dots) under obsNum = 200 and varying maskObs. For each maskObs, only the repetition that exhibits the lowest median of percent bias is visualized. Source data are provided as a Source Data file.

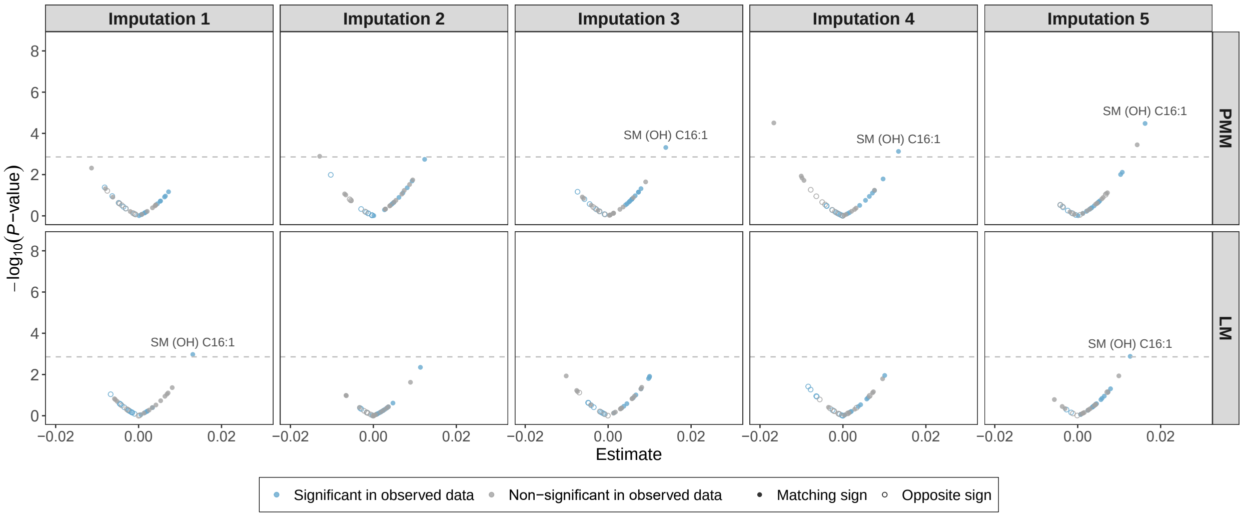

Supplementary Figure 9 The age-associated metabolites identified from each individual imputation (Imputation 1 to 5) of methods PMM and LM. The evaluation is performed on $\mathcal{D}^{\text{test}}_{v=\text{v2},t=\text{t2}}$ ($N = 417$) of the KORA metabolomics dataset, under obsNum = 0. 18 significant metabolites ($P < 0.05/36$) identified from the observed data are shown in blue. Replicated metabolites from the imputed data (obsNum = 0) are marked with labels. Solid dots represent variables where the observed and imputed data have matching signs for the estimate, while hollow dots represent mismatched signs. Source data are provided as a Source Data file.

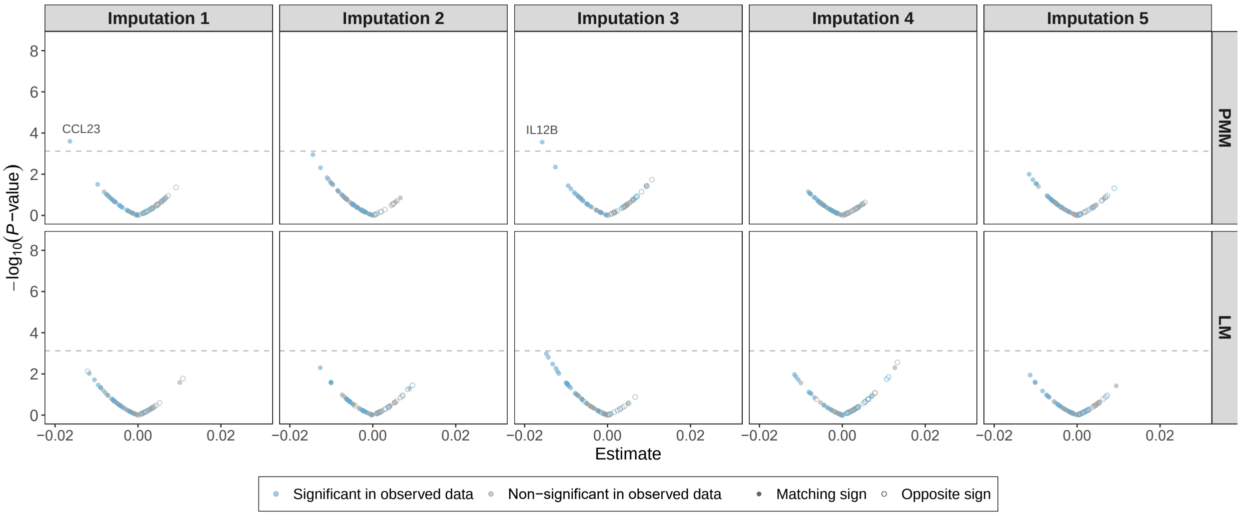

Supplementary Figure 10 The eGFR-associated proteins identified from each individual imputation (Imputation 1 to 5) of methods PMM and LM. The evaluation is performed on $\mathcal{D}^{\text{test}}_{v=\text{v2},t=\text{t2}}$ ($N = 212$) of the KORA multi-omics dataset, under obsNum = 0. 28 significant metabolites ($P < 0.05/66$) identified from the observed data are shown in blue. Replicated metabolites from the imputed data (obsNum = 0) are marked with labels. Solid dots represent variables where the observed and imputed data have matching signs for the estimate, while hollow dots represent mismatched signs. Source data are provided as a Source Data file.

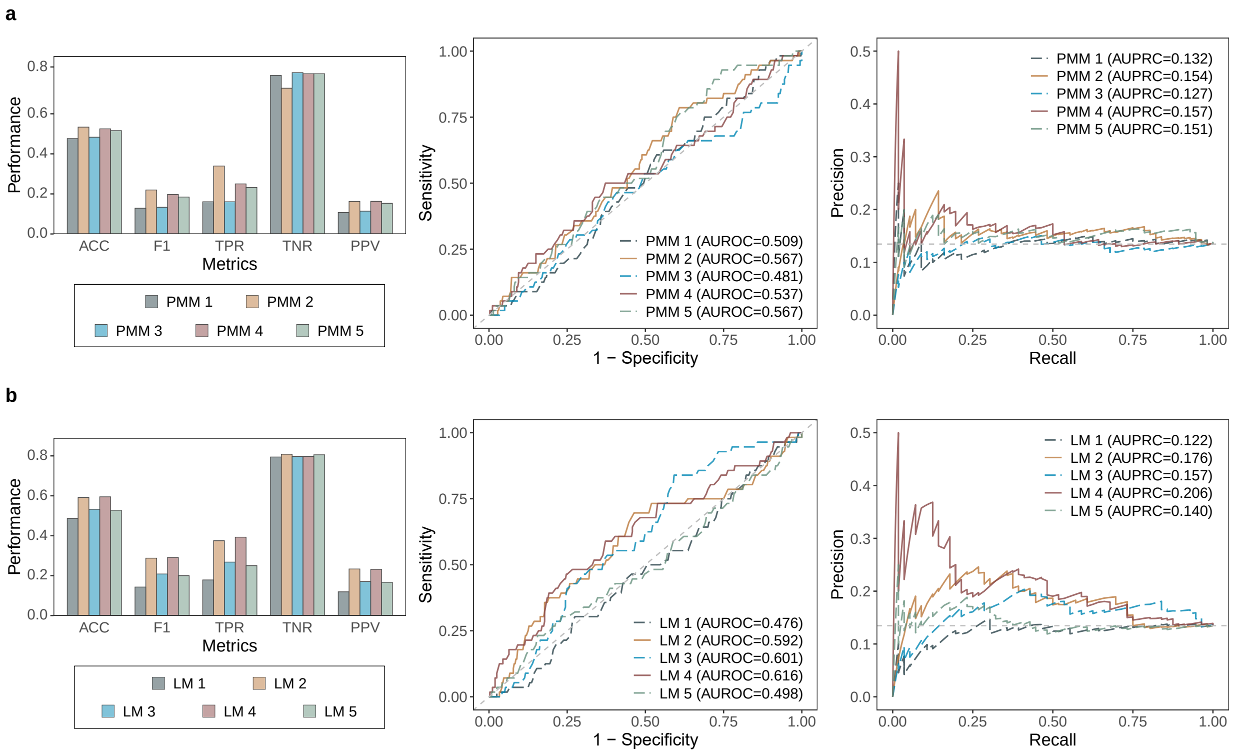

Supplementary Figure 11 The performance of CKD prediction on the KORA metabolomics dataset using each of the 5 multiple imputations produced by methods PMM (**a**) and LM (**b**). Methods are evaluated on $\mathcal{D}^{\text{test}}_{v=\text{v2},t=\text{t2}}$ ($N = 416$, $N_{\text{positive}} = 56$, $N_{\text{negative}} = 360$), under obsNum = 0. PMM 1 to 5 and LM 1 to 5 indicate different individual imputations from PMM and LM, respectively. Models are trained using the BRF algorithm with identical hyperparameters and evaluated using LOOCV. The barplot (left) shows multi-metric performance. The dashed lines in the ROC (middle) and PR (right) curves represent the performance of a hypothetical model with no predictive capability. Source data are provided as a Source Data file.

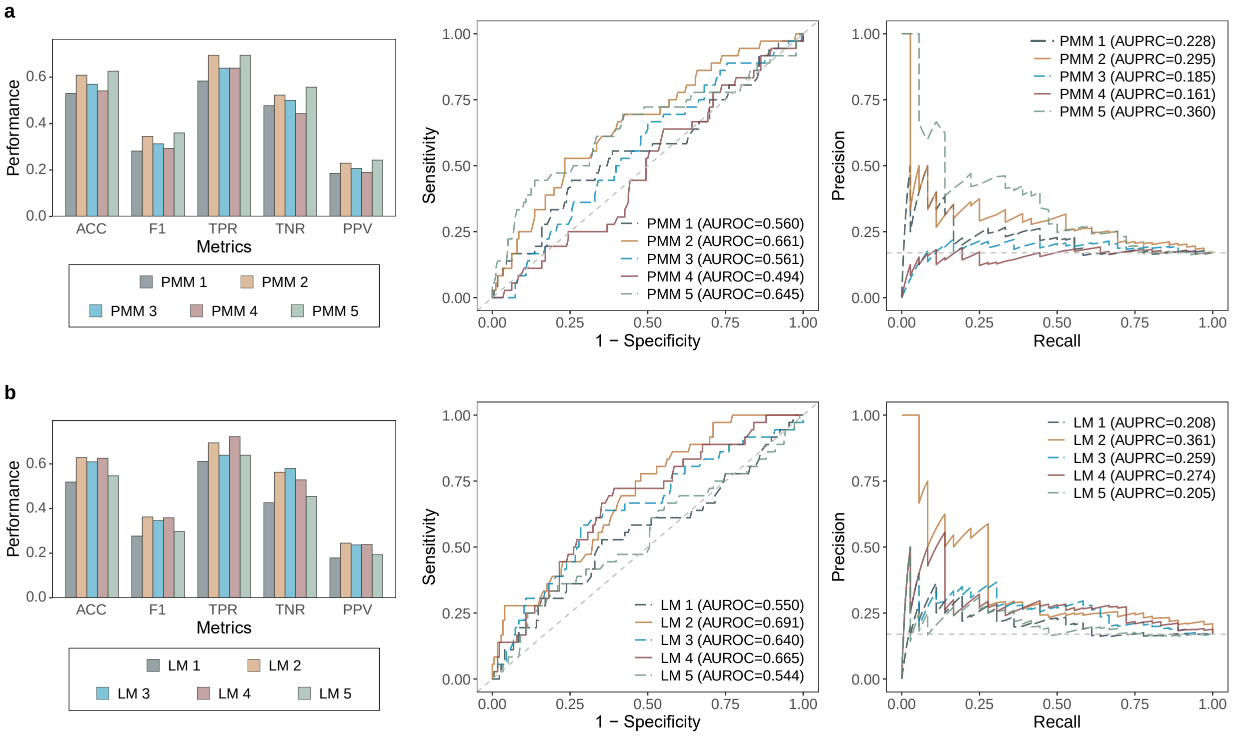

Supplementary Figure 12 The performance of CKD prediction on the KORA multi-omics dataset using each of the 5 multiple imputations produced by methods PMM (**a**) and LM (**b**). Methods are evaluated on $\mathcal{D}^{\text{test}}_{v=\text{v2},t=\text{t2}}$ ($N = 212$, $N_{\text{positive}} = 36$, $N_{\text{negative}} = 176$), under obsNum $= 0$. PMM 1 to 5 and LM 1 to 5 indicate different individual imputations from PMM and LM, respectively. Models are trained using the BRF algorithm with identical hyperparameters and evaluated using LOOCV. The barplot (left) shows multi-metric performance. The dashed lines in the ROC (middle) and PR (right) curves represent the performance of a hypothetical model with no predictive capability. Source data are provided as a Source Data file.

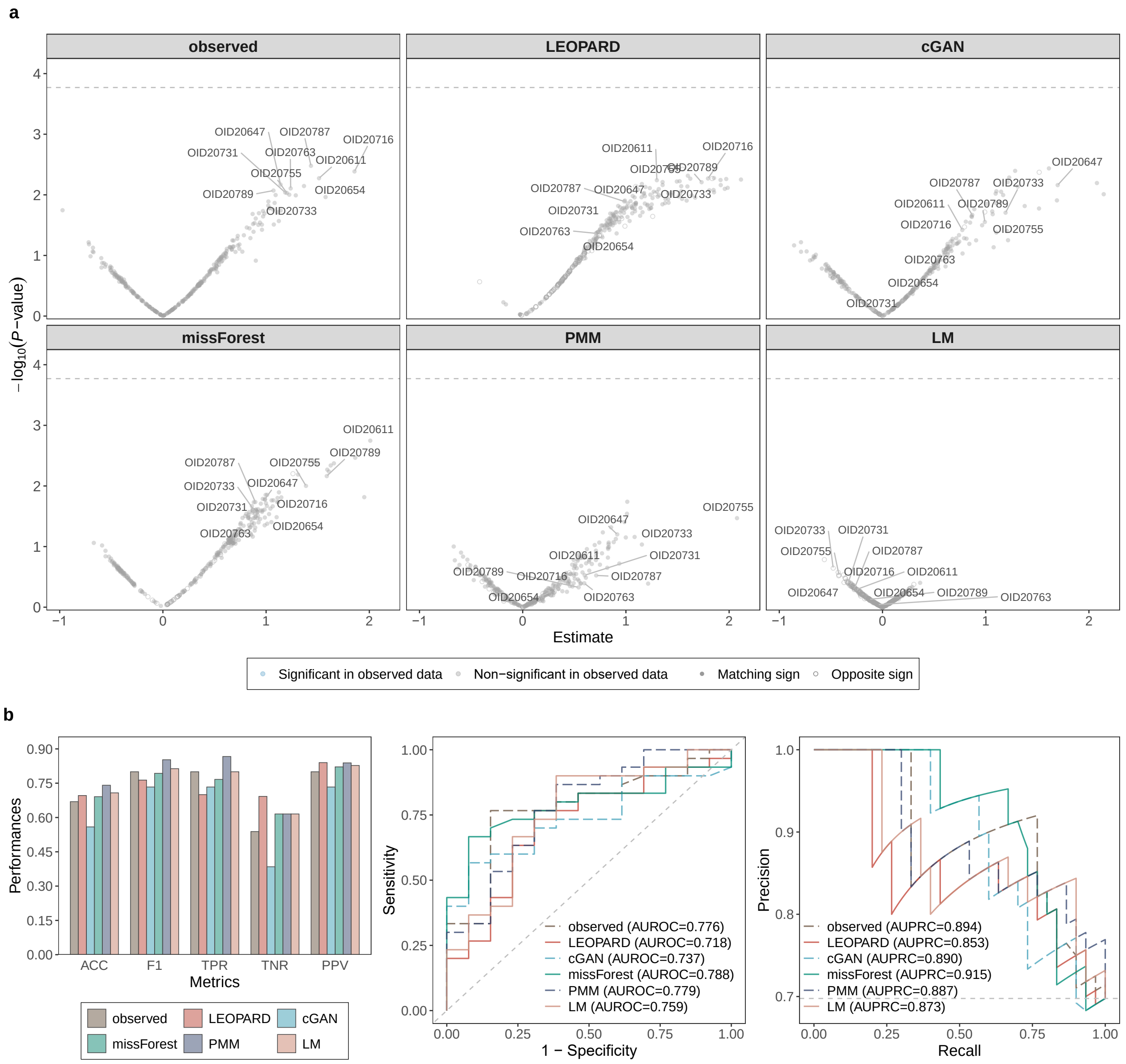

Supplementary Figure 13 **a**, Volcano plots show neutralization level-associated proteins detected from observed and imputed data (obsNum = 0) corresponding to $\mathcal{D}^{\text{test}}_{v=\text{v2},t=\text{t2}}$ ($N = 43$). No significant proteins ($P < 0.05/295$) are identified in the observed data and imputed data. The labels on the plots indicate the top 10 proteins with the lowest P-values. **b**, Neutralization level prediction evaluated on $\mathcal{D}^{\text{test}}_{v=\text{v2},t=\text{t2}}$ ($N = 43$, $N_{\text{positive:HighLevel}} = 30$, $N_{\text{negative:LowLevel}} = 13$), under obsNum = 0. Models are trained using the BRF algorithm with identical hyperparameters and evaluated using LOOCV. The barplot (left) shows multi-metric performance. The dashed lines in the ROC (middle) and PR (right) curves represent the performance of a hypothetical model with no predictive capability. Source data are provided as a Source Data file.

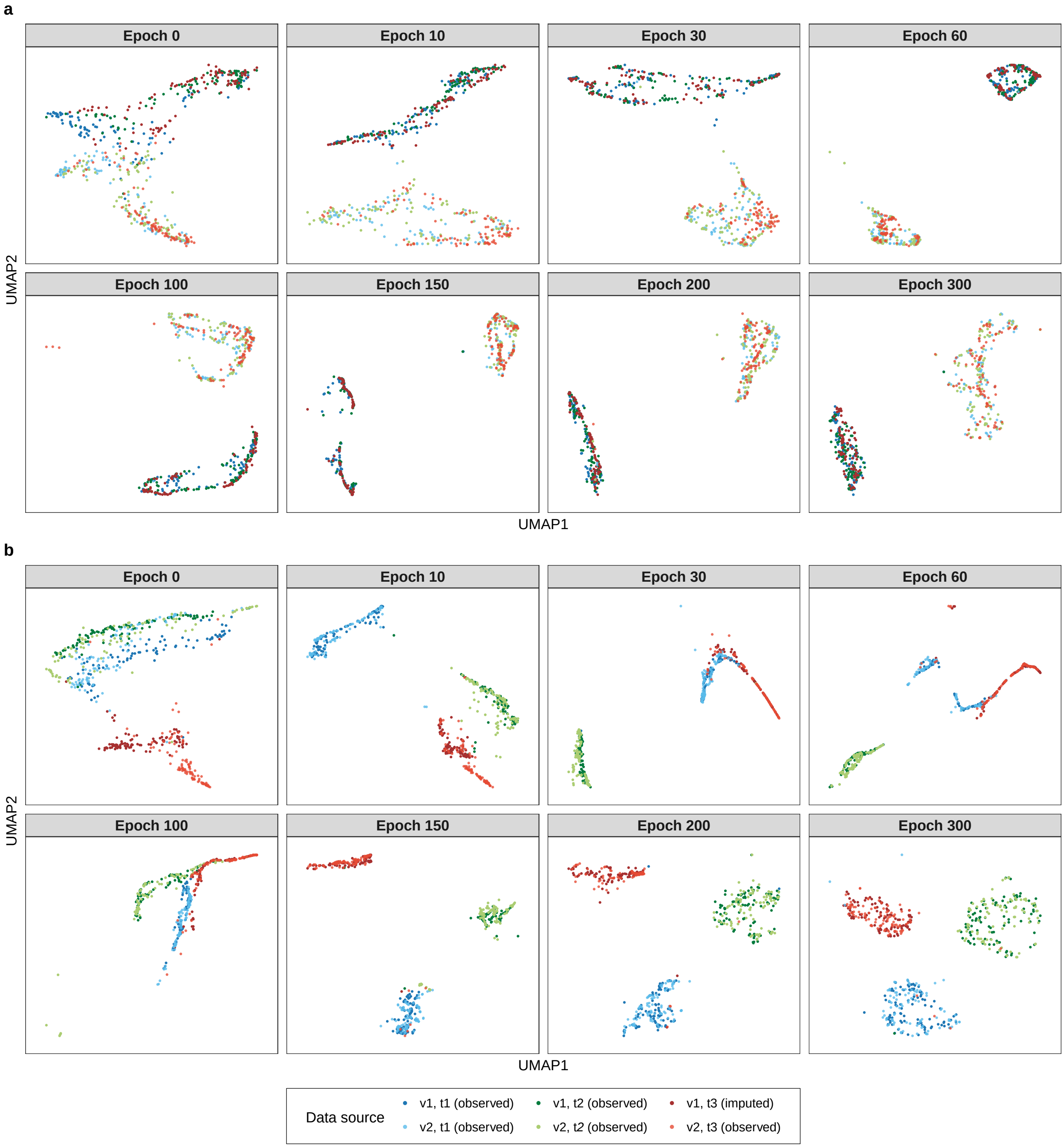

Supplementary Figure 14 a-b, UMAP embeddings of content (**a**) and temporal (**b**) representations at various training epochs for the Extended KORA metabolomics dataset's validation set. Representations encoded from data of v1 and v2 (shown as dark- and light-colored dots) at timepoints t1, t2, and t3 (depicted by blue, green, and red dots) are visualized. The data of v1 at t3 are imputed data produced at each training epoch, while the other data correspond to observed samples in the validation set. The plot shows clusters of similar representations factorized from different data sources. Source Data are provided as a Source Data file.

a

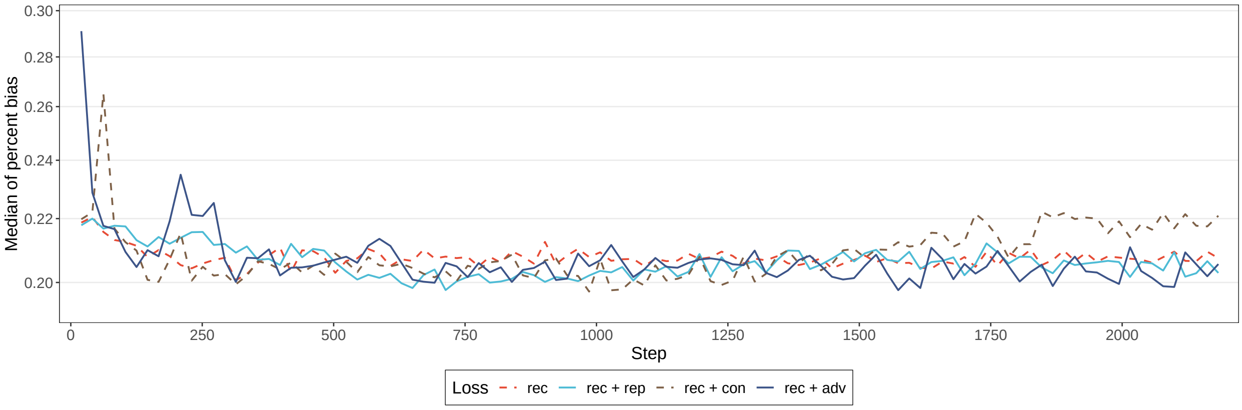

b

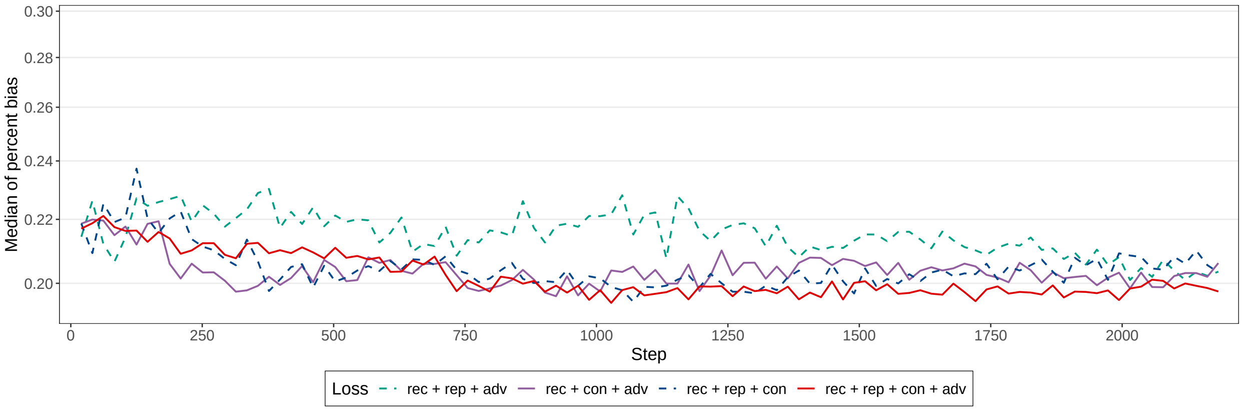

Supplementary Figure 15 The ablation test evaluates the contribution of four losses: reconstructive loss (rec), representation loss (rep), contrastive loss (con), and adversarial loss (adv). Grid search strategy is used to determine the optimal weights for different losses, and the median percent bias computed from the validation set is used to quantify the performance. The network layers are consistent during the evaluation. The plot shows the performance of reconstruction loss combined with (**a**) a single loss and (**b**) multiple losses, using the optimal weight of each loss. The weight for rec is fixed at 1, and the weights for the other losses vary across 0.01, 0.05, 0.1, 0.5, and 1. The optimal weights for different losses in each combination are: rec + rep (w_{rep}=0.01), rec + con (w_{con}=0.01), rec + adv (w_{adv}=0.5), rec + rep (w_{rep}=0.01) + adv (w_{adv}=1), rec + con (w_{con}=0.01) + adv (w_{adv}=1), rec + rep (w_{rep}=0.5) + con (w_{con}=0.01), rec + rep (w_{rep}=0.1) + con (w_{con}=0.1) + adv (w_{adv}=1). Source data are provided as a Source Data file.

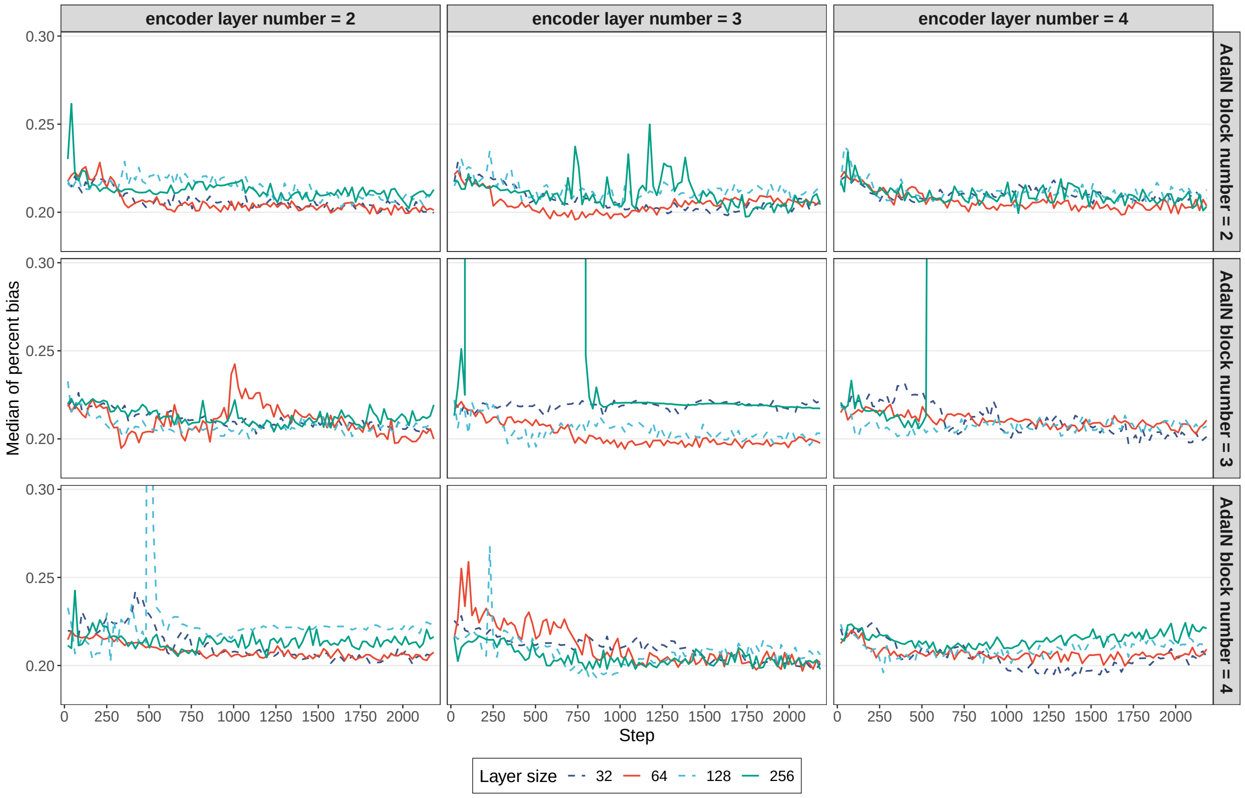

Supplementary Figure 16 Grid search strategy is used to determine the optimal layer number for the content and temporal encoders, AdaIN block number for the generator, and size for each layer. The median percent bias computed from the validation set is used to quantify the performance. The model achieves the lowest median percent bias with a setting of three layers for the encoders, each containing 64 neurons, and three AdaIN blocks for the generators, each including a 64-neuron layer. Source data are provided as a Source Data file.

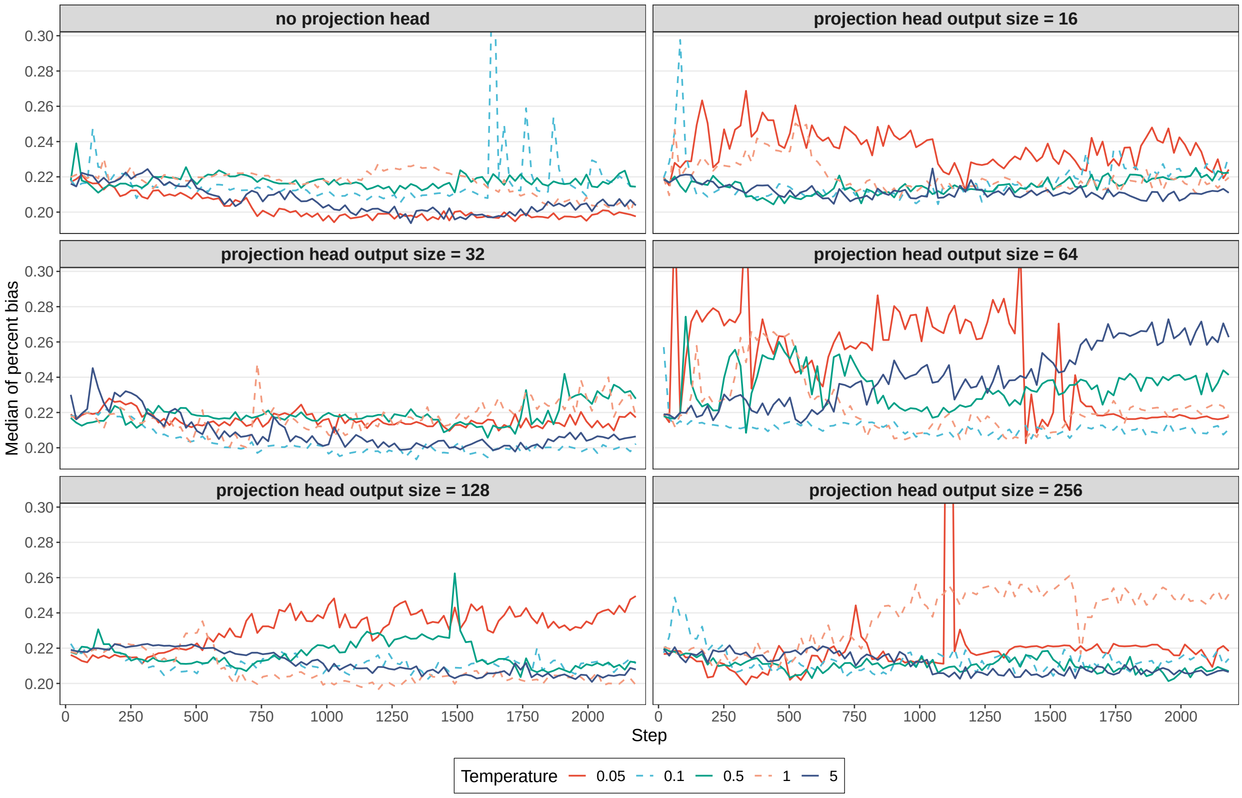

Supplementary Figure 17 Based on the hyperparameters determined in the previous experiments, we further fine-tuned the projection head and temperature used in our contrastive loss. We use grid search to evaluate the performance of LEOPARD without a projection head and with a projection head of different output sizes. The temperature varies across 0.05, 0.1, 0.5, 1, 5, 10, and 30. Based on our experiments, LEOPARD is trained with a temperature of 0.05 without using a projection head. Source data are provided as a Source Data file.

Supplementary Table 1 Method performance of CKD prediction on KORA metabolomics dataset

Method	Sensitivity	Specificity	Precision	Balanced Accuracy	F1 Score	AUROC	PRRPC
observed	0.643	0.800	0.333	0.721	0.439	0.831	0.474
PMM	0.125	**0.897**	0.159	0.511	0.140	0.543	0.186
LM	0.161	0.903	0.205	0.532	0.180	0.590	0.166
missForest	0.268	0.744	0.140	0.506	0.184	0.486	0.135
cGAN	0.268	0.789	0.165	0.528	0.204	0.555	0.177
LEOPARD	**0.571**	0.747	**0.260**	**0.659**	**0.358**	**0.719**	**0.268**

Bold indicates the highest performance among imputation methods.

Supplementary Table 2 Method performance of CKD prediction on KORA multi-omics dataset

Method	Sensitivity	Specificity	Precision	Balanced Accuracy	F1 Score	AUROC	PRRPC
observed	0.778	0.778	0.418	0.778	0.544	0.862	0.593
PMM	**0.667**	0.494	0.212	0.580	0.322	0.631	0.282
LM	0.611	0.562	0.222	0.587	0.326	0.665	0.339
missForest	0.583	0.562	0.214	0.573	0.313	0.589	0.230
cGAN	0.500	0.545	0.184	0.523	0.269	0.551	0.198
LEOPARD	**0.667**	**0.665**	**0.289**	**0.666**	**0.403**	**0.725**	**0.435**

Bold indicates the highest performance among imputation methods.

Supplementary Table 3 Adjusted P-values of percent bias from imputation method comparison

Dataset	obsNum	Bonferroni adjusted P-value (LEOPARD as the reference)			
		cGAN	missForest	PMM	LM/GLMM
MGH COVID proteomics	0	1.62e-14 (***)	1.55e-36 (***)	9.36e-10 (***)	1.08e-16 (***)
	25	2.38e-08 (***)	4.00e-32 (***)	1.23e-13 (***)	1.61e-49 (***)
	50	1.30e-11 (***)	5.08e-41 (***)	3.39e-18 (***)	1.78e-49 (***)
	100	1.62e-14 (***)	2.00e-43 (***)	2.00e-3 (**)	1.69e-49 (***)
KORA metabolomics	0	0.48e-1 (*)	1.13e-1 (ns)	4.08e-06 (***)	8.84e-06 (***)
	50	4.36e-05 (***)	1.16e-09 (***)	1.16e-10 (***)	4.64e-10 (***)
	100	0.18e-1 (*)	6.40e-09 (***)	1.16e-10 (***)	4.64e-10 (***)
	200	1.33e-04 (***)	1.97e-08 (***)	1.16e-10 (***)	4.64e-10 (***)
KORA multi-omics	0	1.74e-1 (ns)	1.000 (ns)	6.68e-05 (***)	4.44e-05 (***)
	50	6.84e-06 (***)	3.45e-08 (***)	7.04e-12 (***)	6.72e-12 (***)
	100	1.02e-1 (ns)	3.32e-08 (***)	7.04e-12 (***)	6.72e-12 (***)
	200	7.92e-11 (***)	2.14e-10 (***)	6.72e-12 (***)	6.72e-12 (***)
