# Supplementary material for: LEOPARD: missing view completion for multi-timepoint omics data via representation disentanglement and temporal knowledge transfer
Source: Nat Commun. 2025 Apr 6;16:3278. doi: 10.1038/s41467-025-58314-3 (PMC11972361; doi:10.1038/s41467-025-58314-3)
Supplement: Supplementary file 2 — Reporting Summary [file 41467_2025_58314_MOESM2_ESM.pdf]

nature portfolio

Corresponding author(s): Rui Wang-Sattler

Last updated by author(s): Feb 26, 2025

Reporting Summary

Nature Portfolio wishes to improve the reproducibility of the work that we publish. This form provides structure for consistency and transparency in reporting. For further information on Nature Portfolio policies, see our Editorial Policies and the Editorial Policy Checklist.

Statistics

For all statistical analyses, confirm that the following items are present in the figure legend, table legend, main text, or Methods section.

n/a	Confirmed	
☐	☒	The exact sample size (*n*) for each experimental group/condition, given as a discrete number and unit of measurement
☐	☒	A statement on whether measurements were taken from distinct samples or whether the same sample was measured repeatedly
☐	☒	The statistical test(s) used AND whether they are one- or two-sided<br>*Only common tests should be described solely by name; describe more complex techniques in the Methods section.*
☒	☐	A description of all covariates tested
☐	☒	A description of any assumptions or corrections, such as tests of normality and adjustment for multiple comparisons
☐	☒	A full description of the statistical parameters including central tendency (e.g. means) or other basic estimates (e.g. regression coefficient) AND variation (e.g. standard deviation) or associated estimates of uncertainty (e.g. confidence intervals)
☐	☒	For null hypothesis testing, the test statistic (e.g. *F*, *t*, *r*) with confidence intervals, effect sizes, degrees of freedom and *P* value noted<br>*Give P values as exact values whenever suitable.*
☒	☐	For Bayesian analysis, information on the choice of priors and Markov chain Monte Carlo settings
☐	☒	For hierarchical and complex designs, identification of the appropriate level for tests and full reporting of outcomes
☒	☐	Estimates of effect sizes (e.g. Cohen's *d*, Pearson's *r*), indicating how they were calculated

Our web collection on statistics for biologists contains articles on many of the points above.

Software and code

Policy information about availability of computer code

Data collection

No software was used for data collection. We constructed the datasets from the sources described in the Data Availability Statement.

Data analysis

Scripts of LEOPARD has been deposited on GitHub (https://github.com/HAN-Siyu/LEOPARD).

The development of LEOPARD was performed based on Python 3.9.15. The following packages were used: numpy (1.21.5), pandas (1.3.5), scikit-learn (1.0.2), pytorch (1.11.10), pytorch_lightning (1.6.4), tensorboard (2.10.0), imbalanced-learn (0.10.1).

The data preprocessing, imputation evaluation, statistical analysis, and visualization were performed under R 4.2.2, with the following packages: TIGERr (1.0.0), mice (3.14.0), missForest (1.5), ggplot2 (3.4.3), umap (0.2.10.0), ggsci (3.0.0), ggrepel (0.9.3), precrec (0.14.1), caret (6.0-93).

For manuscripts utilizing custom algorithms or software that are central to the research but not yet described in published literature, software must be made available to editors and reviewers. We strongly encourage code deposition in a community repository (e.g. GitHub). See the Nature Portfolio guidelines for submitting code & software for further information.

Data

Policy information about availability of data

All manuscripts must include a data availability statement. This statement should provide the following information, where applicable:

- Accession codes, unique identifiers, or web links for publicly available datasets
- A description of any restrictions on data availability
- For clinical datasets or third party data, please ensure that the statement adheres to our policy

The MGH COVID data, published by the original authors, have been deposited to Mendeley Data (http://dx.doi.org/10.17632/nf853r8xsj). For reproducibility, the dataset we constructed from the MGH study and the data we used for visualization can be obtained via LEOPARD's GitHub repository (https://github.com/HAN-Siyu/LEOPARD).

The KORA data are governed by the General Data Protection Regulation (GDPR) and national data protection laws, with additional restrictions imposed by the Ethics Committee of the Bavarian Chamber of Physicians to ensure data privacy of the study participants. Therefore, the data cannot be made freely available in a public repository. However, researchers with a legitimate interest in accessing the data may submit a request through an individual project agreement with KORA via the online portal (https://www.helmholtz-munich.de/en/epi/cohort/kora). Upon receipt of the request, the data access committee will review the application and, subject to approval, provide the researcher with a data usage agreement. The expected timeframe for processing requests and the duration of data access vary depending on the project and are determined by the data access committee. Researchers will receive this information upon submission of their request.

Research involving human participants, their data, or biological material

Policy information about studies with human participants or human data. See also policy information about sex, gender (identity/presentation), and sexual orientation and race, ethnicity and racism.

Reporting on sex and gender	No participants were recruited specifically for this study. Details of the data we used can be found in their publications.
Reporting on race, ethnicity, or other socially relevant groupings	No participants were recruited specifically for this study. Details of the data we used can be found in their publications.
Population characteristics	No participants were recruited specifically for this study. Details of the data we used can be found in their publications.
Recruitment	No participants were recruited specifically for this study. Details of the data we used can be found in their publications.
Ethics oversight	No participants were recruited specifically for this study. We used data from the MGH COVID study and KORA cohort. MGH COVID proteomics data are freely available for all investigators. We obtained the KORA data through an individual project agreement with KORA via the online portal (https://www.helmholtz-munich.de/en/epi/cohort/kora).

Note that full information on the approval of the study protocol must also be provided in the manuscript.

Field-specific reporting

Please select the one below that is the best fit for your research. If you are not sure, read the appropriate sections before making your selection.

☒ Life sciences ☐ Behavioural & social sciences ☐ Ecological, evolutionary & environmental sciences

For a reference copy of the document with all sections, see nature.com/documents/nr-reporting-summary-flat.pdf

Life sciences study design

All studies must disclose on these points even when the disclosure is negative.

Sample size	We constructed datasets with 218 samples from the MGH COVID study, 2085 and 1062 samples from the KORA (F4-FF4) and KORA (S4-F4) studies. We used a random subset of 80% of the data for training and validation, and held out the remaining 20% of the data for evaluation. The sample sizes were determined by the original studies, and all samples that passed QC were included in this study. Recognizing that many other omics datasets may not have as large a sample size as those used here, LEOPARD was evaluated across various sample sizes to ensure its applicability and robustness in diverse scenarios.
Data exclusions	We only used the samples that are available at the timepoints we investigated (timepoints D0 and D3 for the MGH COVID proteomics dataset; timepoints F4 and FF4 for the KORA metabolomics dataset; timepoints S4 and F4 for the KORA multi-omics dataset). For KORA-derived dataset, data from the participants who withdrew data usage consent were also excluded.
Replication	The data from the MGH COVID study and the data we used for visualization can be obtained from LEOPARD's GitHub repository. In the repository, we also provided a jupyter notebook for reproducing the results and plots we reported in our paper. Replication for biological experiments or discovery is not applicable for this computational study.
Randomization	The randomization of the data measurement was performed by the original data administration team, and details can be found in the corresponding publications. In this computational study, training, validation, and test sets were split randomly.

Blinding | This is a computational study, and blinding is not applicable to the experiments in this study.

Reporting for specific materials, systems and methods

We require information from authors about some types of materials, experimental systems and methods used in many studies. Here, indicate whether each material, system or method listed is relevant to your study. If you are not sure if a list item applies to your research, read the appropriate section before selecting a response.

Materials & experimental systems

n/a	Involved in the study
☒	☐ Antibodies
☒	☐ Eukaryotic cell lines
☒	☐ Palaeontology and archaeology
☒	☐ Animals and other organisms
☒	☐ Clinical data
☒	☐ Dual use research of concern
☒	☐ Plants

Methods

n/a	Involved in the study
☒	☐ ChIP-seq
☒	☐ Flow cytometry
☒	☐ MRI-based neuroimaging

Plants

Seed stocks | Np plant data used in this study.

Novel plant genotypes | Np plant data used in this study.

Authentication | Np plant data used in this study.
